# Is my study system good enough? A case study for identifying maternal effects

**DOI:** 10.1002/ece3.2124

**Published:** 2016-04-20

**Authors:** Anna Marie Holand, Ingelin Steinsland

**Affiliations:** ^1^ Department of Mathematical Sciences Centre for Biodiversity Dynamics NTNU NO‐7491 Trondheim Norway

**Keywords:** DIC, INLA, (generalized) linear mixed model (animal model), maternal effects, simulation study

## Abstract

In this paper, we demonstrate how simulation studies can be used to answer questions about identifiability and consequences of omitting effects from a model. The methodology is presented through a case study where identifiability of genetic and/or individual (environmental) maternal effects is explored. Our study system is a wild house sparrow (*Passer domesticus*) population with known pedigree. We fit pedigree‐based (generalized) linear mixed models (animal models), with and without additive genetic and individual maternal effects, and use deviance information criterion (DIC) for choosing between these models. Pedigree and R‐code for simulations are available. For this study system, the simulation studies show that only large maternal effects can be identified. The genetic maternal effect (and similar for individual maternal effect) has to be at least half of the total genetic variance to be identified. The consequences of omitting a maternal effect when it is present are explored. Our results indicate that the total (genetic and individual) variance are accounted for. When an individual (environmental) maternal effect is omitted from the model, this only influences the estimated (direct) individual (environmental) variance. When a genetic maternal effect is omitted from the model, both (direct) genetic and (direct) individual variance estimates are overestimated.

## Introduction

I have a biological hypothesis I want to test for my favorite study system. Is my data set large enough and does it have enough structure to verify my hypothesis? Is the test I use appropriate? And if I leave out important terms in my models what happens with the estimates of the other parameters?

Most quantitative biologists working with natural populations are in this situation of doubt regularly. In this paper, we describe through a case study how simulation studies can be used to answer some of these questions.

Even though simulations are an established methodology (Ripley 1987), it is not common practice in evolutionary biology for natural populations, examples of simulations studies based on natural populations are found in Charmantier and Réale (2005); Kruuk and Hadfield (2007); Morrissey et al. (2007); Hadfield (2008); Hadfield et al. (2011); Larsen et al. (2014).

In this paper, we provide guidelines on how to set up a relevant simulation study for pedigree‐based models for a case study based on a study system of a natural insular population of house sparrows (*Passer domesticus*) (see Ringsby et al. 2002; Jensen et al. 2008; Pärn et al. 2009; Hagen et al. 2013; Baalsrud et al. 2014; Holand et al. 2015; Nossen et al. 2016, and references therein). This case study is based on the same data set as in Holand et al. (2013) and the pedigree is available. We want to know if maternal effects are identifiable for this study system. Further, we want explore the consequences when maternal effects are present, but left out of the model.

Maternal effects have been found to be important in animal breeding (e.g., in mammals Bradford 1972; Koch 1972) for both selection response (Willham 1980; Meyer, 1992, 1997) and predicting rates of inbreeding (Rönnegård and Woolliams 2003).

Maternal effects are present when an individual's trait is not only influenced by its own genes (inherited from the mother and father) and individual (environmental) effects, but also directly by its mother's genes and/or individual effects. These effects can be interpreted as a case of social effects (a common environment effect for all offspring of a mother) (Willham 1963; Bijma et al. 2007; Kruuk and Hadfield 2007). If there is a maternal genetic effect, this will contribute to the heritable variation and may influence adaption and evolution of the trait (Kirkpatrick and Lande 1989; Mousseau and Fox 1998; Wolf et al. 1998; Wolf 2003). However, as identifying maternal effects requires a large amount of data and certain pedigree structures, it has not been much studied in an evolutionary context (Reale et al. 1999; Kruuk et al. 2002; Wilson et al. 2005; Kruuk and Hadfield 2007). How the population structure affect the estimation of maternal effects has been studied (Thompson 1976; Willham 1980; Robison 1981; Meyer , 1992, 1997; Kruuk and Hadfield 2007).

A pedigree‐based (generalized linear) mixed model often called the animal model can be used to identify different genetic and environmental (individual) effects, including maternal effects (Lynch and Walsh 1998; Sorensen and Gianola 2002). Maternal effects are included as a random effect of the identity of each individual's mother in an animal model.

We apply Bayesian animal models (Sorensen and Gianola 2002) and model choice using difference in deviance information criterion (DIC) (Spiegelhalter et al. 2002). The ability of DIC to distinguish between models has been questioned (Claeskens and Hjort 2008; Fong et al. 2010; Gelman et al. 2014). Therefore, we want to validate DIC's ability to choose between models with and without maternal effects for our study system, as well as to find a threshold of difference in DIC to apply. Holand et al. (2013) showed that for this study system (direct) additive genetic effect is identifiable using the difference in DIC.

The simulation study methodology is not tied to these choices of inference and models. The same protocol could be used for maximum likelihood estimation with AIC for model selection. In practices, a simulation study requires the ability to do fast simulations and fast inference. This is available for Bayesian animal models using integrated nested laplace approximations (INLA) (Rue et al. 2009; Steinsland and Jensen 2010; Holand et al. 2013).

The aim of the paper was to propose a protocol for testing whether a given study system is structured enough to accurately identify random effects, for example, maternal effects. The R‐code is available (see *Data accessibility* section).

## Materials and Methods

### Study system

To ensure that our case study is realistic it is based on the pedigree and missing structure of a study system of house sparrow (*Passer domesticus*) populations on six islands off the coast of Helgeland, Northern Norway (66${}^{\circ }$N, 13${}^{\circ }$E). The pedigree spans over seven generation, it consists of $n_{p}\;=\;3574$ individuals and observations are available for our focus trait (bill depth) for $n_{d}\;=\;1025$ of the individuals in the pedigree ($n_{d}<n_{p}$). The study system was used in Holand et al. (2013), and we refer to it and references therein for more in depth descriptions.

### Animal models

To model direct additive genetic and maternal effects, we use an animal model:

(1) $$y_{i}\;=\;\beta_{0}+a_{i}+m_{m(i)}+p_{m(i)}+\epsilon_{i},$$

where $y_{i}$ are the observed trait for individual *i*, $i\;=\;1,...,n_{d}$. $\beta_{0}$ is the intercept, $a_{i}$ is the (additive direct) genetic effect for individual *i*, $m_{m(i)}$ is the additive maternal genetic effect of individual *i*'s mother (*m*(*i*) is the index of the mother of individual *i*). $a_{i}$ and $m_{m(i)}$ are modeled as random structured effects. $p_{m(i)}$ is the maternal individual effect that affect individual *i* and $\epsilon_{i}$ is the (direct) individual effect for individual *i*. $p_{m(i)}$ and $\epsilon_{i}$ are modeled as an independent identical distributed (i.i.d.) effects. Further, each of them ($a_{i}$ and $m_{m(i)}$, and $\epsilon_{i}$ and $p_{m(i)}$) are assumed to be independent. For the population, the additive genetic effect is assumed to follow a Gaussian distribution

$$a|A,\sigma_{a}^{2}∼N\left(0,\sigma_{a}^{2}A\right),$$

where ***A*** is the relationship matrix (see e.g., Lynch and Walsh 1998; Sorensen and Gianola 2002), given by the pedigree, and $\sigma_{a}^{2}$ is the additive genetic variance. Similarly, the maternal genetic effects are assumed to follow

$$m|A,\sigma_{m}^{2}∼N\left(0,\sigma_{m}^{2}A\right),$$

where $\sigma_{m}^{2}$ is the maternal genetic variance. Both individual effects are assumed to be independent and Gaussian; $p∼N(0,\sigma_{p}^{2}I)$, where ***I*** is the identity matrix and $\sigma_{p}^{2}$ is the maternal individual variance, and $\epsilon ∼N(0,\sigma_{\epsilon }^{2}I)$, where $\sigma_{\epsilon }^{2}$ is the individual (direct) variance. Individual effects are often referred to as environmental effects. To complete the Bayesian modeling, priors are assigned to parameters. The variances $\sigma_{a}^{2}$, $\sigma_{m}^{2}$, $\sigma_{p}^{2}$, and $\sigma_{\epsilon }^{2}$ are given *InvGamma*(0.5, 0.5) priors, and $\beta_{0}$ is assigned a flat prior.

In this paper, we use four different animal models, denoted M1–M4. The first model, M1, is an animal model without any maternal effects. M2 and M3 are extensions of M1, including only one of the maternal effects; the genetic maternal effect is included in M2 and the individual maternal effect in M3. M4 is the full model specified in (eq. (1)). These models are summarized in Table 1.

**Table 1:** Models and parameter values used in the simulation studies

Model	*M*1 $y_{i}\;=\;\beta_{0}+\;a_{i}+\epsilon_{i}$	*M*2 $y_{i}\;=\;\beta_{0}+a_{i}+\;m_{m(i)}+\epsilon_{i}$	*M*3 $y_{i}\;=\;\beta_{0}+a_{i}+\;p_{m(i)}+\epsilon_{i}$
Parameter			
$\sigma_{a}^{2}$	0.6	(0.6, 0.5, 0.4, 0.3, 0.2, 0.1, 0)	0.6
$\sigma_{m}^{2}$	–	(0, 0.1, 0.2, 0.3, 0.4, 0.5, 0.6)	–
$\sigma_{p}^{2}$	–	–	(0, 0.1, 0.2, 0.3, 0.4)
$\sigma_{\epsilon }^{2}$	0.4	0.4	(0.4, 0.3, 0.2, 0.1, 0)

### Simulation studies

Traditionally, hypothesis tests are performed for a parameter. Under the null hypothesis, the parameter has a specific value, often zero. Based on a test statistic (often the estimator of the parameter), its sampling distribution and a chosen significant level *α*, we reject the null hypothesis if the probability of getting a more extreme test statistic is less than *α*.

If we do not know the sampling distribution, we can find the critical value by simulations: For each simulation *s*, we sample a data set from the model when $H_{0}$ is true, and calculate the test statistics. We repeat this *S* times, and the (empirical) distribution of the corresponding *S* test statistics is an approximation to our samplings distribution from which we can find critical values of interest. An important property of a test is its power. The power of a test is the probability to reject the null hypothesis when it is not true, that is, to correctly reject $H_{0}$. The power function can be obtained by performing simulations studies for a set of values for the parameters. The proportion of the simulated data sets that are rejected is an estimate of the corresponding power.

#### Model selection and simulations

There are two basic questions we want to answer by simulation studies: (1) Are maternal effects identifiable for our study system? and (2) What is the effect of not including a maternal effect in the model when it is present?

The question about identifiability can be set up in a hypothesis setting. For example, the null hypothesis is the model without maternal effects (M1 in Table 1), and the alternative hypothesis include genetic maternal effects (M2 in Table 1):

(2) $$H_{0}:M1\;y_{i}\;=\;\beta_{0}+a_{i}+\epsilon_{i}$$

(3) $$H_{1}:M2\;y_{i}\;=\;\beta_{0}+a_{i}+m_{m(i)}+\epsilon_{i}$$

We want to find when we can reject $H_{0}$ and conclude with $H_{1}$ based on (simulated) data. To compare the models, we use difference in DIC, Δ*DIC*. If the difference is above a critical value $C_{\Delta DIC}$ we conclude that the $H_{1}$ is true, and we have identified a maternal effect.

Now several choices arise, and we provide suggestions for how to approach these using simulations. Different versions of the generic simulation algorithm below will be used.

#### Simulation algorithm

**Step 1:** Set parameters.

**Step 2:** Simulate *S* new data sets, $y_{s}$, *s* = 1, 2,…*S* according to $H_{0}$ and/or $H_{1}$.

    • Set NA values according to the missing structure of the data set.

**Step 3:** For each data set $y_{s}$, fit model(s) according to $H_{0}$ and/or $H_{1}$, and calculate and/or store relevant quantities.

#### Q1: Which critical value $C_{\Delta DIC}$ should I use to choose between models?

The difference in DIC between the two models is calculated as $\Delta DIC\;=\;DIC\left(H_{0}\right)-DIC\left(H_{1}\right)$. We suggest that $C_{\Delta DIC}$ is based on a chosen significance level *α* and calculated as the corresponding quantile $C_{\Delta DIC}$ of Δ*DIC* based on simulations. In step 2 in the simulation algorithm, we sample according to the model under $H_{0}$. In step 3 models, under both $H_{0}$ and $H_{1}$ are fitted, and $\Delta DIC_{s}\;=\;DIC\left(H_{0}\right)-DIC\left(H_{1}\right)$ is calculated and stored.

We choose a significance level, for example, *α* = 0.05. The appropriate limit for rejecting $H_{0}$ ($C_{\Delta DIC}$) is then calculated from the 95% quantile of the empirical simulated distribution of Δ*DIC*.

#### Q2: Is my study system good enough to identify maternal effects?

The power of a test is the probability that the $H_{0}$ is rejected when $H_{1}$ is true. If $H_{0}$ is rejected, we have identified a maternal effect. The power is a function of the magnitude of the effect, that is, $\sigma_{m}^{2}$ for the test in (eq. (3)). Hence, the question about identifiability translates into: *How do I calculate the power function of the hypothesis test?* To find the power for a specific magnitude of the maternal effect (e.g., a specific value of $\sigma_{m}^{2}$), we can use simulations. In step 2 of the algorithm data sets are simulated from $H_{1}$, and in step 3 both models ($H_{0}$ and $H_{1}$) are fitted and $\Delta DIC_{s}$ calculated and stored. The power is then estimated by the proportions of the $\Delta DIC_{s}$ that are larger than $C_{\Delta DIC}$. The power function can be estimated by doing similar simulations for a range of values for the maternal variance.

#### Q3: What are the consequences of omitting a maternal effect from the model when it is present?

To answer this question, we can do simulations similar to those we used to find the power function. For a specific value of the maternal variance, data sets are simulated from the model under $H_{1}$ (step 2), and in step 3 the model under $H_{0}$ is fitted. The posterior of the parameters (or some of its summary statistics, e.g., posterior mean and 95% credibility interval) are stored. From the posterior means, we have the sampling distribution of the (other) variance parameters when maternal effects are present in the system, but not in the model.

#### Q4: Do my estimates behave well?

If we fit the same model as we have simulated from several times, we want our estimates (i.e., posterior mean) to be close to centered around the true parameter and that the credibility interval is a good quantification of the uncertainty. In statistical terms, we want our estimator to be (close to) unbiased, that is, when the experiment is repeated (many simulations), the mean of the estimates approaches the true parameter. Further, we want the credibility interval to have the right coverage, that is, to be (close to) having the property of a frequentist confidence interval: In the long run, the true parameter should be in the 95% confidence interval in 95% of the simulations. We can check our estimators by simulations. In step 2, we simulate from a model, say $H_{1}$. In step 3, the same model is fitted, that is, $H_{1}$, and posterior quantities such as that posterior mean, median, and the 95% credibility interval are stored. Afterward we can compare the mean of the posterior means with the true parameter values. Further, the coverage can be found by finding the proportions of the credibility intervals that contain the true parameter value.

#### How should I set the parameter values such that my simulation study is relevant?

The results obtained from the simulation study depend on the values chosen for the parameters. How should we choose the values for the parameters so that they are realistic to the trait in question? We suggest that the true data set first is fitted to the simplest model we consider, that is, an animal model without maternal effects (M1) for our study. Denote the estimated variances from this model ${\hat{\sigma }}_{a,M1}^{2}$ and ${\hat{\sigma }}_{\epsilon ,M1}^{2}$. These estimates can be used to guide the parameter choices in the simulation studies. Both because of computational cost, and to be able to interpret results, it is practical to make some restrictions. We suggest that the genetic and individual effects should be hold constant and equal to ${\hat{\sigma }}_{a,M1}^{2}$ and ${\hat{\sigma }}_{\epsilon ,M1}^{2}$, respectively. For model M1 and M4 that means that values for $\sigma_{a}^{2}$ and $\sigma_{m}^{2}$ are chosen such that $\sigma_{a}^{2}+\sigma_{m}^{2}\;=\;{\hat{\sigma }}_{a,M1}^{2}$ and for model M3 and M4 $\sigma_{\epsilon }^{2}+\sigma_{p}^{2}\;=\;{\hat{\sigma }}_{\epsilon ,M1}^{2}$.

#### Simulation settings in the house sparrow case study

**Figure 1:**
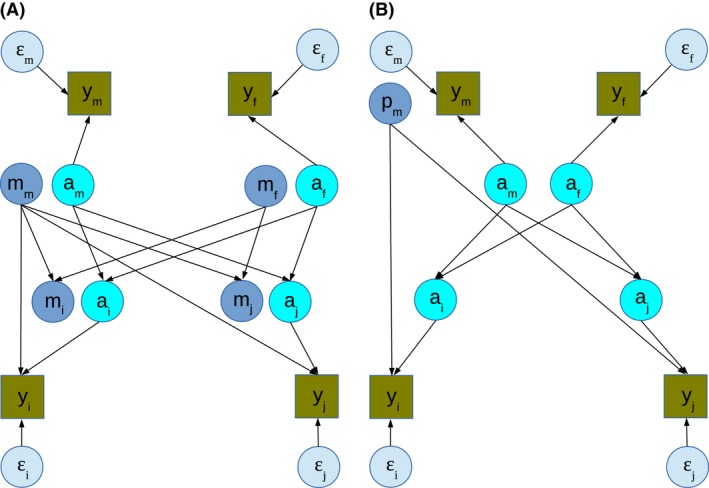
Graphical presentation of the models used in simulation studies (S1) and (S2). (A) Model *M*2. (B) Model *M*3. The models in (A) and (B) are given in Table 1. Direct individual effects are indicated by *ε*, direct additive genetic effects are indicated by *a*, maternal genetic effects are indicated by *m*, maternal individual effects are indicated by *p*, phenotypic trait values are indicated by *y*, subscript *i* and *j* indicate individual *i* and *j*, respectively, subscript *m* indicate mother, subscript *f* indicate father, phenotypes are visualized by green squares and random effects are visualized by blue ellipses.

We show results for two simulation studies. Simulation study 1 (S1) is according to the tests; $H_{0}:M1$ versus $H_{1}:M2$ (no maternal effects vs. genetic maternal effects), and simulation study 2 (S2) is according to the test $H_{0}:M1$ versus $H_{1}:M3$ (no maternal effects vs. individual maternal effects). See Figure 1 for a graphical description of model *M*2 and *M*3. We also explored what happens when we go from one to two maternal effects, that is, $H_{0}:M2$ versus $H_{1}:M4$ and $H_{0}:M3$ versus $H_{1}:M4$, but these results are not presented. Only Gaussian traits were considered, and all simulations and inference were performed using AnimalINLA R package (Holand and Martino 2016) with sum to zero constraints on the direct and maternal genetic effects, $\sum a_{i}\;=\;0$ and $\sum m_{i}\;=\;0$ (Steinsland and Jensen 2010). For each parameter set, we simulated *S* = 1000 data sets.

We set parameters according to our suggestions for setting parameters in section *How should I set the parameter values such that my simulation study is relevant?*, and set ${\hat{\sigma }}_{a,M1}^{2}\;=\;0.6$ and ${\hat{\sigma }}_{\epsilon ,M1}^{2}\;=\;0.4$, giving us a phenotypic variance $\sigma_{P}^{2}\;=\;1$, and $\beta_{0}\;=\;0$. The phenotypic data are only used for this purpose. Further, a discretization of 0.1 was used for the variance parameters. The parameter values used in the simulation studies S1 and S2 are summarized in Table 1.

## Results

### Simulation study (S1)

In the first simulation study (S1), we consider $H_{0}:M1$ versus $H_{1}:M2$, that is, an animal model without maternal effects versus one with maternal genetic effects.

#### Q1: Which critical value $C_{\Delta DIC}$ should I use to choose between models?

**Figure 2:**
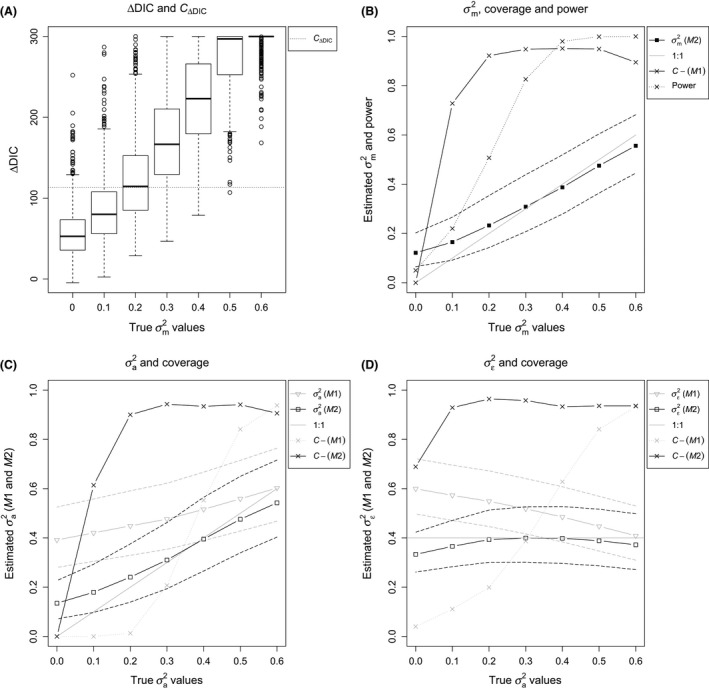
Results from simulation studies (S1). (A) Boxplots of simulated values of Δ*DIC* against the true value of $\sigma_{m}^{2}$ in (M2). $C_{\Delta DIC}\;=\;110$ is indicated (dotted line). (B) Posterior mean (filled squares/solid lines) with mean 95% credible interval (dashed line) for $\sigma_{m}^{2}$ (M2), power of the model selection test (x‘es/dotted line), coverage (C) (x‘es/solid line) of the 95% CI for the posterior mean of $\sigma_{m}^{2}$ (M2). (C) Posterior mean for $\sigma_{a}^{2}$ and mean 95% credible interval (dashed lines) for $\sigma_{a}^{2}$ when fitting (M1) (gray) and (M2) (black), coverage (C) of the 95% CI for the posterior mean of $\sigma_{a}^{2}$ (M1) (x‘s/dotted line, gray) and (M2) (x‘es/solid line, black). (D) Posterior mean of $\sigma_{\epsilon }^{2}$ and mean 95% credible interval (dashed lines) for $\sigma_{\epsilon }^{2}$ when fitting (M1) (gray) and (M2) (black), coverage (C) of the 95% CI for the posterior mean of $\sigma_{\epsilon }^{2}$ (M1) (x‘s/dotted line, gray) and (M2) (x‘es/solid line, black). A 1:1 function of true versus estimated parameter values are indicated in (A), (B), and (C) (gray line).

We first find the critical value $C_{\Delta DIC}$. The sampling distribution for Δ*DIC* when *M*1 is true is given by the boxplot for $\sigma_{m}^{2}\;=\;0$ in Figure 2A. The critical value is found to be $C_{\Delta DIC}\;=\;110$ and is marked by a horizontal dotted line.

#### Q2: Is my study system good enough to identify maternal effects?

Next, we want to find how large the genetic maternal effect has to be to be identified. The sampling distributions for different values of the maternal genetic variances ($\sigma_{m}^{2}$) are visualized by boxplots in Figure 2A, and the corresponding power function can be found in Figure 2B. We see that the maternal genetic variance of $\sigma_{m}^{2}\;=\;0.3$ is needed to get a power of 0.8. Hence, a substantial portion of the genetic effects needs to be maternal (remember $\sigma_{a}^{2}+\sigma_{m}^{2}\;=\;0.6$) to have a high probability to identify it.

#### Q3: What are the consequences of omitting a maternal effect from the model when it is present?

The effect of not including the maternal effects in the model when they are present can be explored in Figure 2C and D (gray lines). From Figure 2C, we find that fitting a model without maternal effects (M1) gives a higher estimate of the additive genetic variance ($\sigma_{a}^{2}$) than the model including additive maternal effects (M2). But not all the genetic variance (they sum to 0.6) are taken up by $\sigma_{a}^{2}$. Indeed, we see from Figure 2D that also the estimated direct individual variance $\sigma_{\epsilon }^{2}$ increases when the maternal effect is high. That is, it seems that excluding the maternal genetic effect from the model causes both the additive (direct) genetic variance estimate ($\sigma_{a}^{2}$) and (direct) individual variance estimate ($\sigma_{\epsilon }^{2}$) to increase.

#### Q4: Do my estimates behave well?

To explore the properties of the variance estimators, we look at the coverage (Fig. 2B) and the mean of posterior means and mean of 2.5% and 97.5% posterior quantiles for model M2. We have biased estimator, when either $\sigma_{m}^{2}$ or $\sigma_{a}^{2}$ is a relative small part of the total genetic variance ($\sigma_{a}^{2}+\sigma_{m}^{2}\;=\;0.6$). This is also found in Holand et al. (2013) for models with only direct additive genetic effects and is due to prior sensitivity. The coverage for $\sigma_{m}^{2}$ has to be 0 for $\sigma_{m}^{2}\;=\;0$ as it is the lower limit of the parameter's domain. We find that also the coverage is poor when one of the genetic effects is small. This might also be due to prior sensitivity.

### Simulation study (S2)

In the second simulation study (S2), we consider $H_{0}:M1$ versus $H_{1}:M3$, that is, an animal model without maternal effects versus one with maternal individual effects.

#### Q1: Which critical value $C_{\Delta DIC}$ should I use to choose between models?

**Figure 3:**
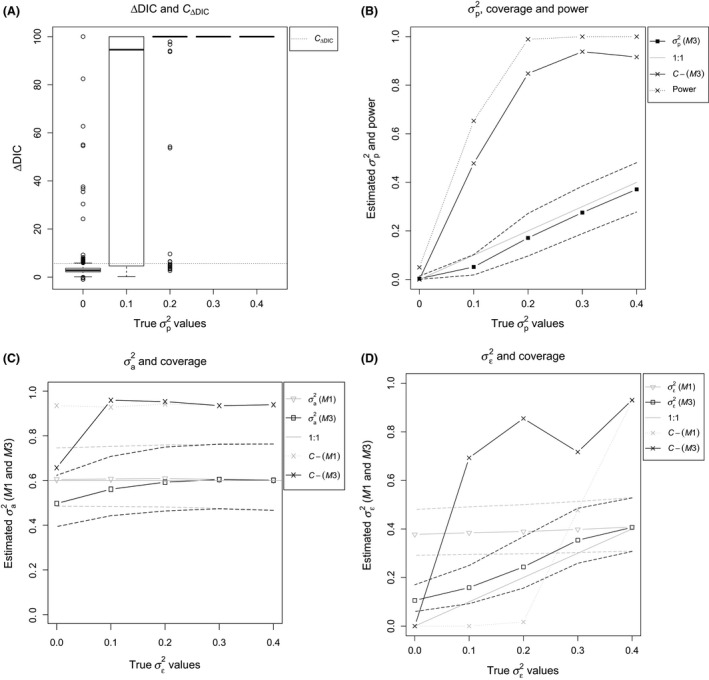
Results from simulation studies (S2). (A) Boxplots of simulated values of Δ*DIC* against the true value of $\sigma_{p}^{2}$ in (M3). $C_{\Delta DIC}\;=\;7$ is indicated (dotted line). (B) Posterior mean (filled squares/solid lines) with mean 95% credible interval (dashed line) for $\sigma_{p}^{2}$ (M3), power of the model selection test (x‘es/dotted line), coverage (C) (x‘es/solid line) of the 95% CI for the posterior mean of $\sigma_{p}^{2}$ (M3). (C) Posterior mean for $\sigma_{a}^{2}$ and mean 95% credible interval (dashed lines) for $\sigma_{a}^{2}$ when fitting (M1) (gray) and (M3) (black), coverage (C) of the 95% CI for the posterior mean of $\sigma_{a}^{2}$ (M1) (x‘s/dotted line, gray) and (M3) (x‘es/solid line, black). (D) Posterior mean of $\sigma_{\epsilon }^{2}$ and mean 95% credible interval (dashed lines) for $\sigma_{\epsilon }^{2}$ when fitting (M1) (gray) and (M3) (black), coverage (C) of the 95% CI for the posterior mean of $\sigma_{\epsilon }^{2}$ (M1) (x‘s/dotted line, gray) and (M3) (x‘es/solid line, black). A 1:1 function of true versus estimated parameter values are indicated in (A), (B), and (C) (gray line).

The results are summarized in Figure 3 in the same way as in for the S1. The critical value is found to be $C_{\Delta DIC}\;=\;7$.

#### Q2: Is my study system good enough to identify maternal effects?

From Figure 3B, we find that the power function increase fast and is 0.6 for $\sigma_{p}^{2}\;=\;0.1$ and close to 1 for $\sigma_{p}^{2}\;=\;0.2$. But, this is still a substantial proportion of the individual variances (remember $\sigma_{\epsilon }^{2}+\sigma_{p}^{2}\;=\;0.4$).

#### Q3: What are the consequences of omitting a maternal effect from the model when it is present?

To evaluate the consequences of not including the maternal individual effects in the model when they are present, we study Figure 3C and D. From Figure 3D, we find that the estimated direct individual variance ($\sigma_{\epsilon }^{2}$) when fitting the model M1 (without maternal effects) is approximately 0.4 ($\sigma_{\epsilon }^{2}+\sigma_{p}^{2}=0.4$) independently of the true maternal individual variance ($\sigma_{p}^{2}$). Further, from Figure 3C, we find that when fitting M1 the additive genetic variance estimate ($\sigma_{a}^{2}$) does not change systematically as a function of the individual maternal variance $\sigma_{m}^{2}$. In this situation, it seems as the omitted maternal individual effects are taken up by the direct individual effects only.

#### Q4: Do my estimates behave well?

To explore the properties of the variance estimators, we look at the coverage (Fig. 3B) and the mean of posterior mean and mean of 2.5% and 97.5% posterior quantiles for model M3. We find that the individual maternal variance estimate is downward biased, while the direct individual variance estimate is upward biased, especially for small values of $\sigma_{\epsilon }^{2}$. This might also cause the poor coverage of $\sigma_{\epsilon }^{2}$ and $\sigma_{p}^{2}$. As for (S1), the coverages are poor when one of the variances is small (Fig. 3B–D). This may be due to prior sensitivity as discussed above. We further notice that the coverage for $\sigma_{a}^{2}$ when fitting the wrong model (M1) is good for all values of $\sigma_{\epsilon }^{2}$. On the other hand, when fitting the correct model (M3) coverage is poor for $\sigma_{a}^{2}$ for small values of $\sigma_{\epsilon }^{2}$. From this we learn that for our study system it is not possible to estimate variance parameters in model M3 precisely, the estimates are biased, and the confidence intervals do not have the right coverage.

The ability to identify and distinguish both genetic and individual maternal effects (M4) can be explored similarly. The results (not presented here) show that this study system neither has the right properties to identify both individual and genetic maternal effects, nor to estimate the maternal variances precisely.

## Discussion

We have used simulation studies to explore and gain understanding of our study system's ability to identify maternal effects as well as the consequences of omitting maternal effects from the model when they are present. We have learned that maternal effects can (only) be identified if they are substantial. For our study system, the genetic maternal effect has to be about half of the (total) genetic to be identified. We also get similar result for individual maternal effect.

We have considered a Gaussian trait and maternal effects. The same methodology can also be used for non‐Gaussian traits, and other effects, for example, additive genetic effects or sex‐linked effects or the consequences of missing not at random (Holand et al. 2013; Larsen et al. 2014; Steinsland et al. 2014).

We have used DIC to choose between models and have found critical values using simulations. This ensures a certain significance level. The use of DIC has been questioned. Our two different tests (S1 and S2) gave very different critical values ($C_{\Delta DIC}=110$ and $C_{\Delta DIC}=7$). This indicates that using one fixed critical value independent of the models compared (e.g., $C_{\Delta DIC}=10$ as performed in Holand et al. (2013)) might give unintended properties. The ability of other model selection criteria of choosing the correct model can be explored with similar simulation studies.

Fitting a model without maternal effects when maternal effects are present will affect the estimated variance parameters. Generally, the total true variance seems to be accounted for. When the maternal individual (environmental) effect is omitted from the model, only the (direct) individual variance seems to be affected, and it is estimated to be the sum of direct and maternal individual effects. This result is in accordance with the findings in Larsen et al. (2014). They performed a simulation study where they simulated a trait with both autosomal and sex‐linked additive genetic effects. They found that when fitting the trait in a model without sex‐linked genetic effect, this gave an estimate of the autosomal genetic variance corresponding to the total amount of (additive) genetic effect in the trait.

When a maternal genetic effect is omitted from the model, it influences estimates of both direct genetic and direct individual variances. The total (genetic and individual) variance are accounted for in the results, but the total amount of genetic variance (direct and maternal) is not always picked up by the estimated (direct) genetic variance. Hence, it is not generally true that all genetic effects are accounted for by a (direct) additive genetic effect.

The simulation studies showed that when at least one of the variance parameters are close to zero, we might have biased estimates, and poor coverage for several of the variance parameters. This indicates prior sensitivity, and we have learned that priors needs to be carefully chosen, and prior sensitivity should be checked.

For complex systems, it is difficult to have an intuition for our study system's ability to identify effects of interest and how omitting effects that are present influence parameter. Simulations studies are a powerful tool in this situation. Fast simulation and inference make simulations studies more attractable. We are able to explore identifiability properties and the consequences of omitting effects from the model for our study system with the models (including priors) and inference method we have chosen.

## Data Accessibility

The house sparrow pedigree and R‐code for performing the simulation studies are archived in the AnimalINLA R package (available at www.r-inla.org).

## Conflict of Interest

None declared.
